# Supplementary material for: An Integrative Process-Based Model for Biomass and Yield Estimation of Hardneck Garlic (Allium sativum)
Source: Front Plant Sci. 2022 Mar 15;13:783810. doi: 10.3389/fpls.2022.783810 (PMC8967357; doi:10.3389/fpls.2022.783810)
Supplement: Supplementary file 1 [file Data_Sheet_1.PDF]

Supplementary Material

1 SUPPLEMENTARY DATA

1.1 Cold Stress

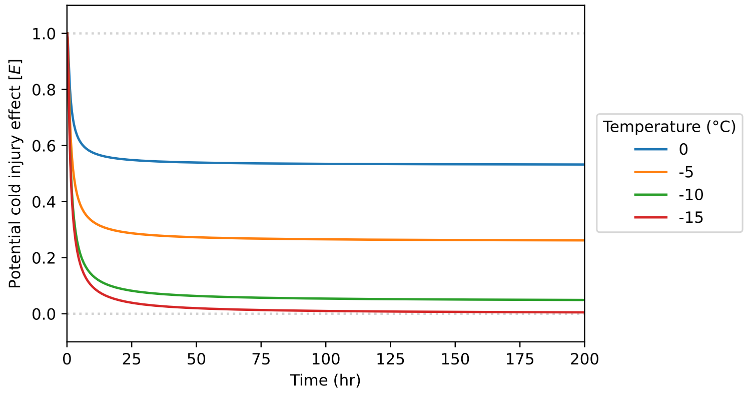

Figure S1. Potential cold injury effect ($E = 1 - \frac{C}{e^{\frac{1}{D}}}$) calculated for a given temperature (T) at a certain time t.

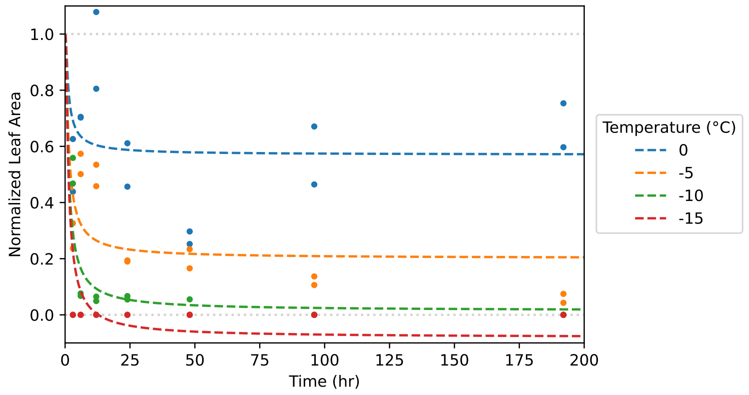

Figure S2. To derive potential cold injury effect (E), a curve of normalized leaf area was fitted with the observations for each temperature treatments. Each curve provided an estimate of apparent cold injury effect (C) for the given temperature.

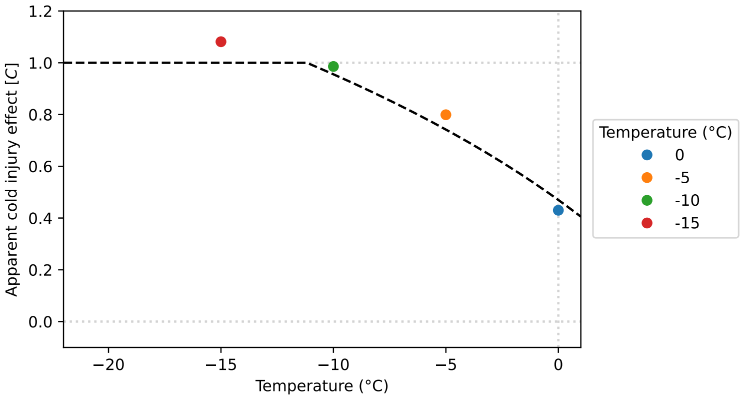

Figure S3. Apparent cold injury effect ($C = \max\left(0, \min\left(\log\left[a \cdot (T - T_{c,i}) + b\right], 1\right)\right)$) fitted with the estimates from Figure S2.

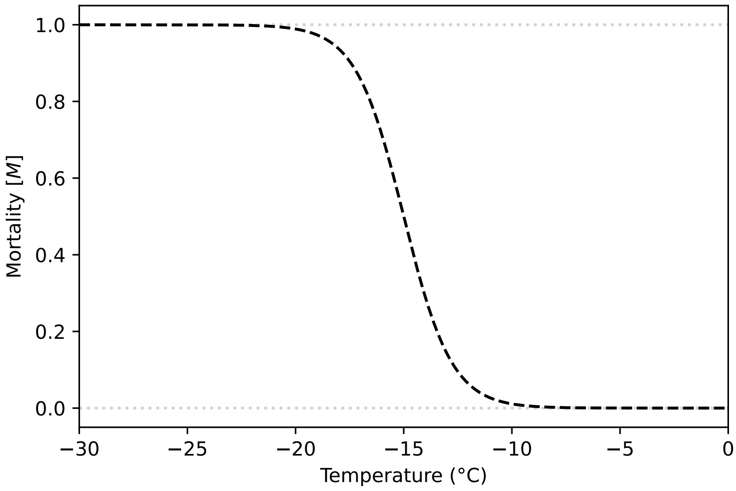

Figure S4. Mortality ($M = \frac{e^{-s(T-T_{c,d})}}{1+e^{-s(T-T_{c,d})}}$) for reducing plant density (PD) due to permanent cold damage.
